# Developing health care provider knowledge, confidence, and cultural sensitivity through resident transgender training: a controlled educational study

**DOI:** 10.1186/s12939-025-02555-7

**Published:** 2025-07-10

**Authors:** Kathie Huang, Almira J. Yang, Lynnetta Skoretz, Anthony Firek, Dhruv Khurana

**Affiliations:** 1 Department of Medicine, Riverside University Health System, 26520 Cactus Avenue, Moreno Valley, CA 92555 USA; 2 Department of Medicine, University of California Riverside School of Medicine, Riverside, CA USA; 3 Department of Medicine, Loma Linda University School of Medicine, Loma Linda, CA USA; 4 Department of Psychiatry and Biobehavioral Sciences, University of California Los Angeles David Geffen School of Medicine, Los Angeles, CA USA; 5 Department of Health Policy and Management, University of California Los Angeles Fielding School of Public Health, Los Angeles, CA USA; 6 Comparative Effectiveness and Clinical Outcomes Research Center (CECORC), Riverside University Health System, Moreno Valley, CA USA

**Keywords:** Transgender, Gender diverse, Graduate medical education, Internal medicine, Gender-affirming care, Health disparities, Curriculum development

## Abstract

**Background:**

Transgender and gender-diverse (TGD) individuals face substantial health disparities as a result of discrimination and poor provider competence in understanding their health needs. Relatively little work has been done studying educational interventions targeted toward increasing residents’ knowledge and ability to treat TGD individuals with sensitivity. We studied the effectiveness of implementing a lecture series on transgender health in preparing internal medicine residents to care for the TGD population.

**Methods:**

Both study and control participants were recruited through their affiliated internal medicine residency programs. The study design was a pre-post controlled educational study. A lecture series was developed at Riverside University Health System as the educational intervention. We used a Transgender Assessment survey developed for the study to determine changes in the residents’ knowledge, self-confidence, and knowledge of barriers to care during the study period from January to June 2022. The data were statistically analyzed to assess the differences between pre- and post- and study and control groups.

**Results:**

Similar demographics were noted between the study and control groups. Compared with the control group, residents in the study group tended to have more exposure to transgender health education prior to the study. Residents in the study group demonstrated increased knowledge and self-confidence after completing the curriculum. The study group's average knowledge score increased from 4.8 to 6.1 post-intervention (*p* = 0.004). Self-confidence scores in providing gender-specific care rose from an average of 13.7 to 17.9 post-intervention (*p* < 0.001). The study group had higher post-intervention scores compared to the control group, particularly in knowledge of gender-affirming therapies (post 4.3 vs. pre 3.4, *p* = 0.01) and self-confidence in providing gender-specific care (post 17.9 vs. pre 12.3, p=0.004). No significant changes were observed in knowledge of barriers to care for both groups.

**Conclusions:**

Our study demonstrates the effectiveness of a curriculum focused on TGD health in improving residents' knowledge and confidence. Further research is needed on the durability of these effects and the curriculum's impact on awareness of barriers to care. Implementing such curricula at other institutions could reinforce educational programs in medical schools to improve provider competence and address the healthcare needs of TGD individuals.

**Supplementary Information:**

The online version contains supplementary material available at 10.1186/s12939-025-02555-7.

## Background

Transgender and gender-diverse (TGD) individuals encounter pervasive discrimination, harassment and stigma that result in minority stress leading to poorer health outcomes compared with their cisgender peers [1–5]. These poorer actual and self-reported outcomes encompass physical and mental health conditions including HIV, cardiovascular disease, alcohol and tobacco use, depression and suicidality [2, 3]. Along with decreased healthcare access, the limited competence of health care providers treating TGD patients is a significant barrier to more equitable care [6]. Problems engaging with standard healthcare settings include the fear of discrimination which is grounded in the experiences reported by members of this population [1, 7, 8]. The critical lack of familiarity among medical trainees and professionals appears ubiquitous and encompasses everything from the fundamentals of how to approach TGD individuals about their gender identity, to their health, mental health, and gender affirmation needs [9–12]. Further research is needed to examine the effect of educational interventions on improving trainees’ ability to treat TGD individuals and recognize the disparities they encounter.

In medical training, the unique physical and mental health needs related to sexual orientation and gender identity are increasingly being addressed at the undergraduate medical education level, but gaps remain in transgender training at the graduate level [13–15]. Curricular content has been developed for internal medicine residents focused on primary care for lesbian, gay, bisexual, and transgender (LGBT) patients [16, 17]. Although such studies have demonstrated promising improvements in knowledge, fewer investigators have focused specifically on the TGD population, with the incumbent risk of their unique problems being overlooked when included in general education about sexual and gender minority health. Existing studies focused on education about the TGD population have generally involved one-time educational interventions for medical students and residents [18–21]. Published outcomes on longitudinal, TGD-focused education are more limited, particularly for internal medicine trainees in a primary care setting [14, 15]. Building learners’ awareness of the barriers to care for this population is also essential to improving health, reducing inequities, and eliminating mistreatment in healthcare. In a recent survey of United States residency program directors, barriers to TGD people accessing medical care comprised a key area that is not being addressed in current graduate medical education [22]. An understanding of the discrimination faced by TGD individuals is fundamental to building cultural sensitivity and humility in trainees, a need that has been recognized as essential to effective training in transgender health care at all levels of medical education [15, 23].

To address these urgent unmet needs, we developed a didactic series on the health of TGD individuals with the purpose of studying its effectiveness on increasing internal medicine residents’ preparedness to care for TGD patients. A lecture series was developed to cover critical healthcare topics, including gender-affirming therapies and preventive care. Using a Transgender Assessment survey, we determined the changes in the residents’ knowledge, self-confidence, and knowledge of barriers to care during the study period from January to June 2022.

## Methods

### Study population

Study participants were resident physicians at Riverside University Health System (RUHS) recruited through their affiliated internal medicine residency programs (RUHS, University of California Riverside, and Loma Linda University). A total of 63 residents were eligible if they participated in continuity clinic and ambulatory didactics at RUHS. The internal medicine continuity clinic is a federally qualified health center (FQHC) that provides primary care to patients with predominantly Medicaid or Medicare (public) insurance or are underinsured. A control group was recruited from internal medicine residents at Loma Linda University, who had their continuity clinic at another FQHC not affiliated with RUHS. We included a control group in order to assess the extent that any observed differences could be attributable to the curriculum in the intervention group. Residents were informed that their participation in the study was voluntary, and no personal identifiers were collected. Informed consent was obtained from all individual participants included in the study. The study was approved by the RUHS institutional review board (11/15/2021, IRB # 1791096).

### Curriculum development and implementation

The intervention consisted of a series of 4 lecture sessions that were developed on the following topics:

1. Introduction to Transgender Care
2. Mental Health Evaluation for TGD Individuals
3. Overview of Medical Therapies
4. Preventive Care for TGD Individuals.

Lectures 1 and 4 were adapted from modules initially developed for the Veterans Affairs Medical Center and were updated using guidelines on gender-affirming medical care published by the University of California San Francisco, Fenway Health, and the World Professional Association for Transgender Health (WPATH) [24–27]. A fifth lecture session addressed fundamental concepts of caring for TGD individuals and was conducted by a speaker invited from WPATH who spoke from lived experience. Except for the latter, the presentations were provided by RUHS faculty in endocrinology, psychiatry and internal medicine who had received prior training in TGD care through fellowship (endocrinology), WPATH courses (endocrinology and internal medicine), and providing gender-affirming care (all). The psychiatry lecturer was an expert in the field of TGD mental health care.

The lectures were presented sequentially over four months. The lectures lasted 45 min to one hour, followed by a brief question-and-answer session. All lectures were broadcast virtually and recorded. Lectures 2, 3, and 4 were conducted in a hybrid format, with the speaker and facilitator being present with a live audience and online. Recordings of each lecture were made available to the study population for viewing if they could not attend at the scheduled time.

### Curriculum assessment

A Transgender Assessment survey was developed for use pre- and post-intervention. The Assessment underwent qualitative content validation by experts on TGD-focused care. A TGD cultural community liaison also reviewed the survey for language appropriateness. Then, the Assessment was piloted in two focus groups of chief residents who had finished internal medicine training during the preceding academic year. Minor revisions to the question wording were made for clarity in response to feedback from the focus groups.

The Transgender Assessment aligned with three domains corresponding to the primary aims of the curriculum in determining improvement in each domain:

1. Residents’ knowledge about transgender care (Knowledge domain)
2. Residents’ self-perceived confidence and competence in transgender care (Self-confidence domain)
3. Residents’ knowledge of barriers that TGD individuals face while obtaining care (Knowledge of Barriers domain).

For the Knowledge portion of the Transgender Assessment, content from the didactic sessions was used to develop a 9-question multiple choice test on three domains:

1. Gender-affirming medical and surgical therapies (5 questions)
2. Screening and preventive care (2 questions)
3. Sexual health (2 questions)

The multiple-choice questions were written per standard testing principles for applying clinical knowledge [28]. Residents received one point for every correct response. No negative marking was associated with the score calculation for the Knowledge Section. Consequently, the knowledge section scores ranged from 0 to 9, where a higher score indicated more knowledge.

We adapted the items in the Knowledge of Barriers and Self-confidence sections from the Sexual Orientation Counselor Competency Scale (SOCCS) version 3, a validated self-assessment of mental health providers’ attitudes regarding their skills, beliefs, and knowledge of affirming transgender mental health services [29, 30]. SOCCS was partly developed to assess provider competency and understanding of challenges facing the TGD population. The Knowledge of Barriers to Care Section (4 items) consisted of four statements with a five-point Likert scale going from “Strongly Disagree” to “Strongly Agree” and a score range from 4 to 20. All statements were presented with a negative sentiment, where a higher value indicated a higher awareness of barriers to care facing TGD individuals. Similarly, the Self-confidence section included five statements with a five-point Likert scale and a score range from 5 to 25. All statements were presented with a positive sentiment, where a higher value indicated more confidence in providing transgender care.

### Data collection and analysis

We used the Survey Monkey^©^ online platform for administering our survey. The Transgender Assessment was made accessible to the study and control groups for one month before the intervention's commencement and one month after the conclusion of the last lecture. Residents were offered a financial incentive of USD 5 for each survey completed. Both the study and control groups received the same pre-education survey. In contrast, the post-education assessment survey differed in that only residents in the study group were asked to indicate if they attended the didactic sessions and which sessions they had completed.

Both pre- and post-surveys assessed the residents’ demographics and status of familiarity with transgender care. The pre- and post-survey responses were not paired due to the lack of a unique identifier. Consequently, we cannot draw any causal conclusions. We dropped surveys without any responses. The highest attainable training level in internal medicine residency is PGY-3; we excluded surveys from residents who reported a training level exceeding PGY-3. In the post-intervention study group, those who responded that they had attended at least one didactic session were included in the analysis. We calculated both intra-group differences, i.e., how the characteristics within each group (study and control) evolved over time (pre- and post-intervention), and inter-group differences, i.e., the changes in study and control groups before intervention and the changes in study and control groups post-intervention to confirm initial equivalence and detect post-intervention divergence. These are provided in Appendix 1 (Supplementary Material 1). The data were analyzed using R v4.3.3 and STATA MP-17 software, with Wilcoxon rank sum, Pearson’s chi-squared, and Fisher’s exact tests of statistical significance being used to test the differences between pre- and post- and study and control groups, where applicable.

## Results

In the study group, 48 out of 63 (76.2%) responses to the Transgender Assessment were received pre-intervention, and 26 out of 63 (41.3%) were received post-intervention. In the control group, 16 out of 48 (33.3%) responses were received pre-intervention, and 10 out of 48 (20.8%) were received post-intervention. After accounting for the exclusion criteria outlined in the Methods section, the total count was 42 for the pre-intervention study group, 21 for the post-intervention study group, 14 for the pre-intervention control group, and 9 for the post-intervention control group. Out of those in the study group who responded to the post-intervention survey, the attendance rate ranged from 75 to 87.5% across the lectures.

Table 1 presents demographics and the residents’ familiarity and interaction with TGD individuals, including their experience in providing transgender care. During the pre-intervention stage, the study sample included more females (52%) and more individuals identifying as straight (95%). The distribution by training level was as follows: 43% PGY 1, 21% PGY 2, and 36% PGY 3 respondents. None of the respondents identified as being transgender or gender diverse. However, 24% of residents personally knew someone who identified as transgender. Similar differences were noted during the post-intervention stage.

**Table 1.**
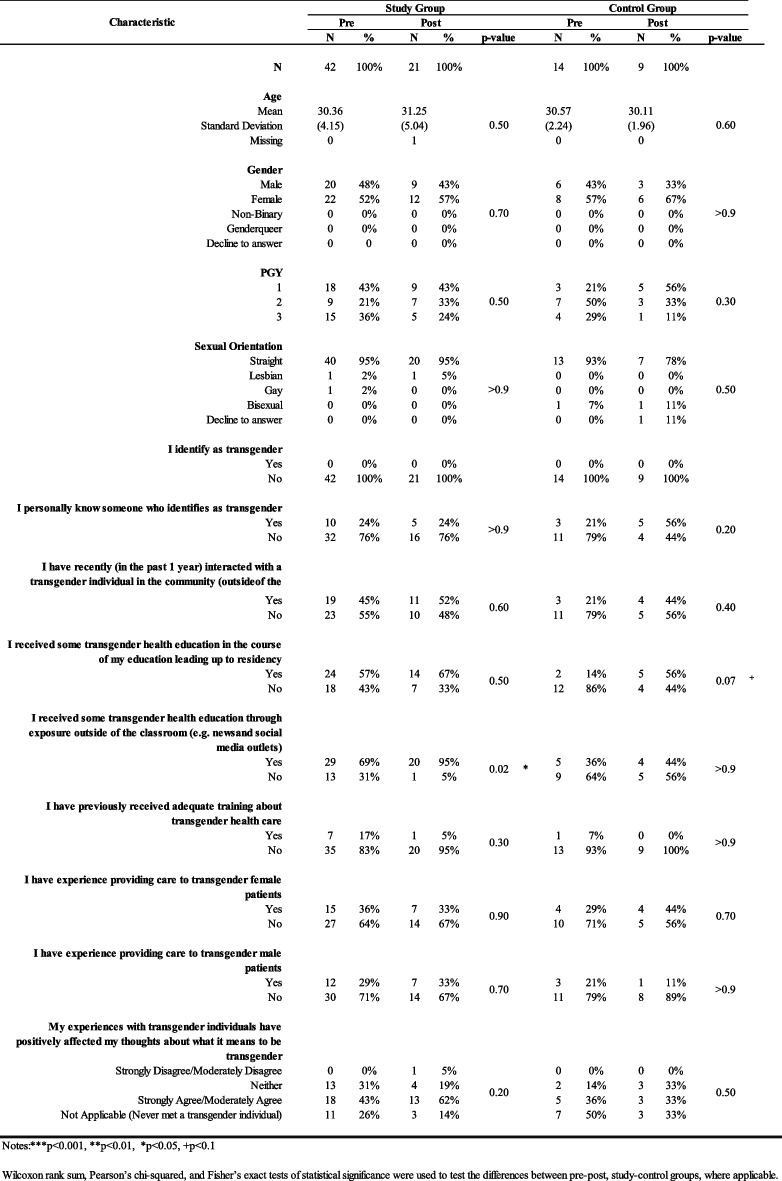
Presents data comparing various demographic characteristics as well as factors related to transgender health education and experience providing care to TGD patients between the study group and the control group, both before and after the intervention. Notes: *p<0.05, **p<0.01, ***p<0.001

None of these demographic characteristics differed significantly between the study and the control group at either stage. However, 57% of the residents in the study group during the pre-intervention stage “received some transgender health education in the course of their education leading up to residency,” compared to only 14% of residents in the control group (*p* < 0.01). Further, 69% of the residents in the study group “received some transgender health education through exposure outside of the classroom (e.g., news and social media outlets)” during the study and leading up to the post-study survey. In contrast, only 36% of the respondents in the control group answered yes to this statement (*p*<0.05) in the same period. It is unknown if the attendance in the didactics may have promoted residents’ exposure outside the classroom.

Based on the results presented in Table 2, for the knowledge section, overall, the study group saw a statistically significant increase from 4.8 (SD = 1.5) to 6.1 (SD = 1.3) before and after the intervention (*p* = 0.004). Within the knowledge section, the study group's average knowledge of Gender Affirming Medical and Surgical Therapies increased significantly from 3.4 (SD = 1.23) to post 4.3 (SD = 1.05) (*p* = 0.01), and average knowledge of Sexual Health increased statistically from 1 (SD = 0.69) to 1.4 (SD = 0.6) (*p* = 0.03). No statistically significant differences were noted in the control group over the same period for any scores. Post-intervention, the study group scored significantly higher than the control group for the Gender Affirming Medical and Surgical Therapies category, with an average score of 3.4 (SD=0.94) compared to 2.6 (SD=1.06), *p*=0.02). No statistically significant results were observed between pre- and post-for either study or control groups for questions related to Knowledge of Barriers, as shown in Table 3.

**Table 2.**
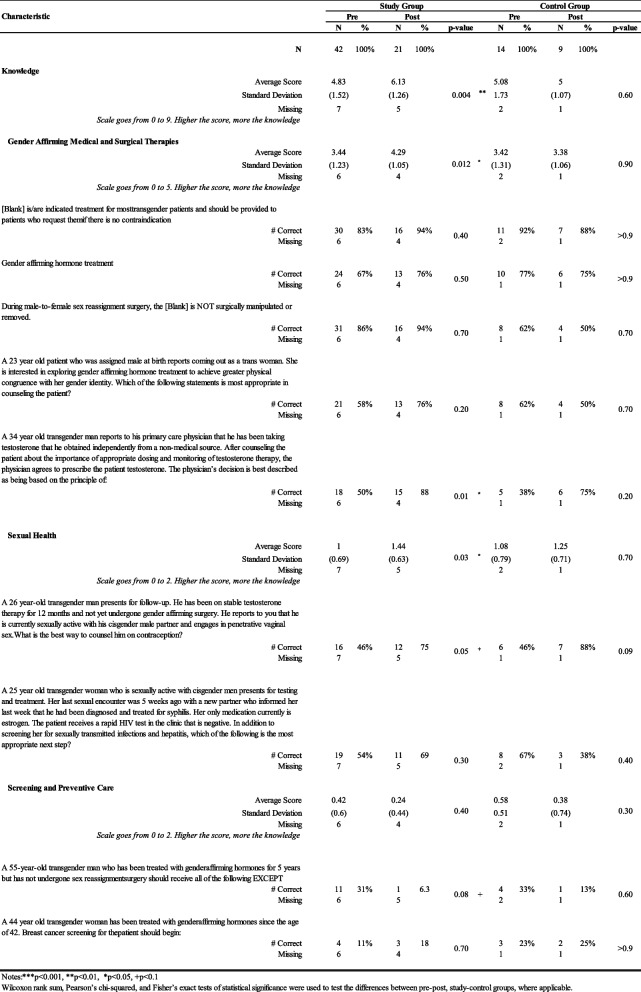
Illustrates the shifts within the study and control groups before and after the intervention, capturing changes in Knowledge over time. Correct responses to specific questions related to transgender healthcare are also listed, showing the percentage of correct answers for each group. Missing data are noted, and the scale for each category is explained where applicable. Notes: *p<0.05, **p<0.01, ***p<0.001

**Table 3.**
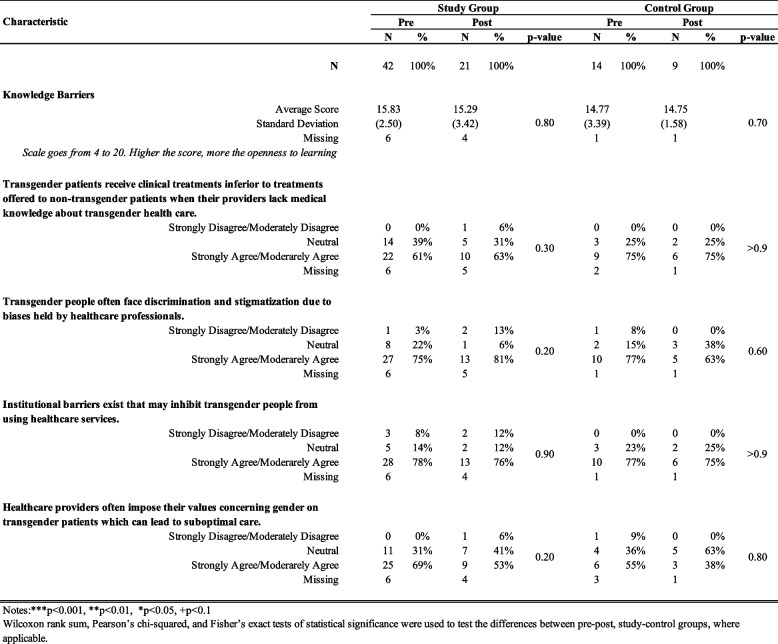
Looks at how the Knowledge of Barriers characteristics changed within the study and control groups before and after the intervention. It tracks shifts over time. The higher the score, the more open they were. The data suggests that there was little change within or between the groups. Further, the table explores participants' attitudes towards transgender healthcare, covering topics like discrimination, stigmatization, institutional barriers, and value imposition by healthcare providers. Notes: *p<0.05, **p<0.01, ***p<0.001

For Self-confidence regarding transgender health, Table 4, Panel I presents the pre-and post-intervention data within the study and control groups. The study group’s average score for Self-confidence increased statistically from 13.7 (SD=3.6) to 17.9 (SD=3.6) post-intervention (*p* < 0.001), indicating a notable enhancement in participants' confidence levels. Specifically, more residents felt “confident to assess the medical needs of transgender patients” (*p* < 0.001), felt “confident to provide gender health-specific care to transgender patients” (*p* = 0.002), and felt “confident assessing when a patient who was assigned female at birth reports identifying as a male since adolescence and requests gender-affirming hormonal therapy” post-intervention (*p* = 0.015). Further, the study group scored higher than the control group regarding Self-confidence post-intervention, with an average of 17.9 (SD = 3.6) compared to 12.3 (SD = 3.4) (*p* = 0.004). Specifically, when compared with the control group post-intervention, more study group participants felt “comfortable communicating with transgender patients” (*p* = 0.059)**,** felt “confident in providing gender health-specific care to transgender patients” (*p* = 0.003), and felt “confident assessing when a patient who was assigned female at birth reports identifying as a male since adolescence and requests gender-affirming hormonal therapy” (*p* = 0.007).

**Table 4.**
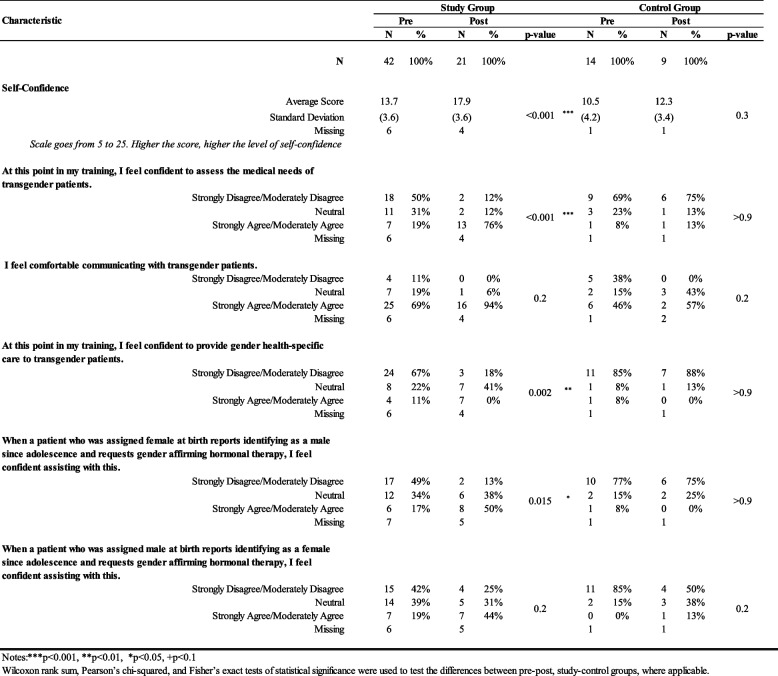
Shows a significant increase in Self-confidence among participants in both study and control groups following the intervention, with the study group exhibiting higher post-intervention confidence levels. Notes: *p<0.05, **p<0.01, ***p<0.001

We ran several regression models described below to further discern the intervention’s impact on the Knowledge, Knowledge of Barriers, and Self-confidence outcomes. For each outcome, we estimated both unadjusted and adjusted regression models: ordered logit for ordinal variables (Likert statements under Knowledge of Barriers and Self-Confidence), logistic regression for binary variables (individual Knowledge questions), and OLS for continuous summary scores. To minimize overfitting with our small sample, covariate selection was limited to the two binary items on which study and control groups differed significantly (Appendix Table 1.1): “I received some transgender health education through exposure outside of the classroom (e.g., news and social media outlets)” and “I received some transgender health education in the course of my education leading up to residency”). All other baseline characteristics showed no statistically significant imbalance and were therefore excluded from the adjusted models. These results are presented in the Appendix as well.

Model estimated:

$${\mathrm Y}_{\mathrm i}={\mathrm\beta}_0+{\mathrm\beta}_1\left({\mathrm{Treat}}_{\mathrm i}\times{\mathrm{Time}}_{\mathrm i}\right)+{\mathrm\beta}_2\;{\mathrm X}_{\mathrm i}+{\mathrm\varepsilon}_{\mathrm i}$$

​Here:

- Y_i_ is the outcome (ordinal, binary, or continuous, depending on the question).
- Treat_i_ equals 1 for lecture participants and 0 for controls.
- Time_i_ equals 1 in the post‐intervention period and 0 at baseline.
- The product Treat_i_×Time_i_ captures the difference‐in‐differences effect (β_1_), i.e., how much more (or less) the treatment group changed over time compared with the control group.
- X_i_ represents the two binary covariates on which groups differed at baseline (1 = yes, 0 = no).
- β_2_ is a vector of coefficients for those covariates.
- ε_i_ is the residual error term.

Based on the regression results in Table 5, in the unadjusted Difference‐in‐Differences ordered‐logit model, the interaction term for “At this point in my training, I feel confident to assess the medical needs of transgender patients” had an odds ratio of 2.31 (SE = 1.21; *p* = 0.12), suggesting that curriculum attendees were more than twice as likely as controls to report a higher confidence level post‐intervention, although this did not reach statistical significance. After adjusting for baseline exposure to transgender health education (outside the classroom and during prior training), the interaction odds ratio increased to 2.54 (SE = 1.15; *p* = 0.04), indicating that, even when accounting for those two covariates, residents who received the lecture were significantly more likely than controls to move into a higher confidence category.

**Table 5.**
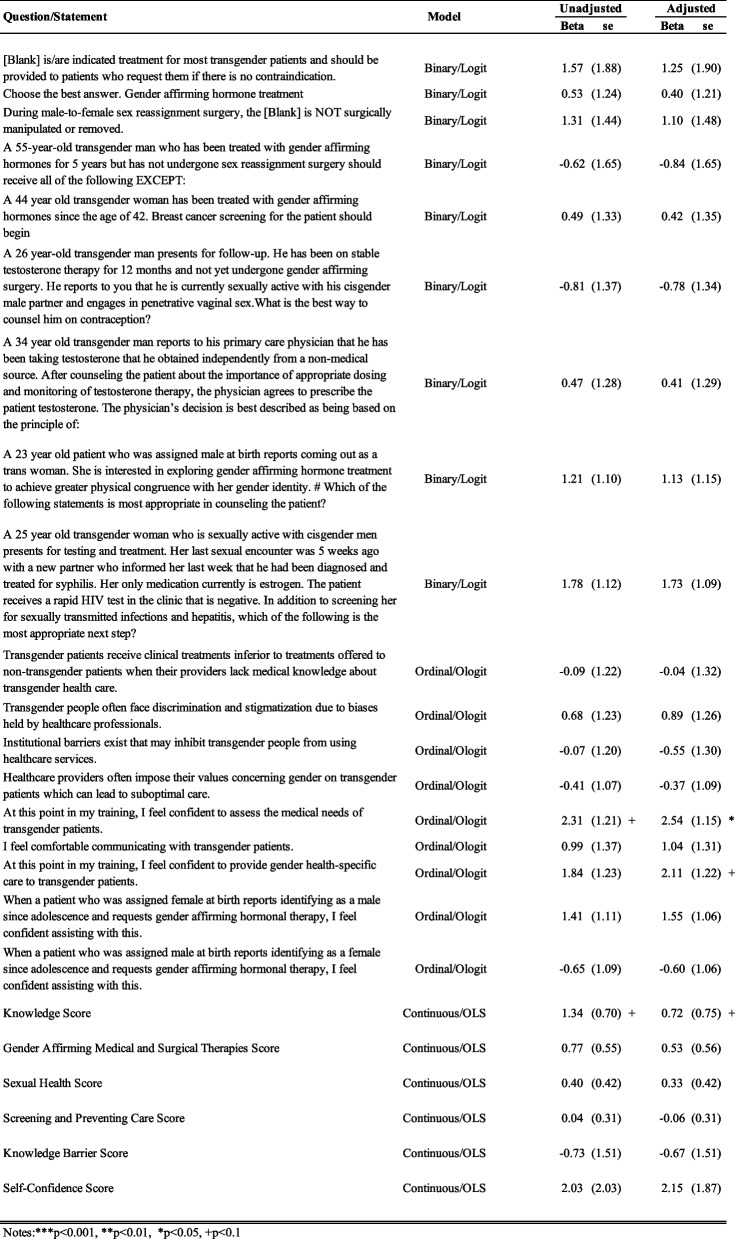
Represents the beta coefficients for the interaction term obtained from unadjusted and adjusted regression models based on the outcome variable as mentioned in the second column. The adjusted coefficients account for the two binary variables ("I received some transgender health education through exposure outside of the classroom (e.g., news and social media outlets)” and “I received some transgender health education in the course of my education leading up to residency”) that were statistically significantly different between the study and control groups at baseline

Similarly, in the unadjusted ordered‐logit model for “I feel confident to provide gender health–specific care to transgender patients,” the Difference-in-Differences estimate yielded an odds ratio of 1.84 (SE = 1.23; *p* = 0.26), indicating a non‐significant 84% increase in the odds of reporting higher confidence among lecture attendees compared with controls. After adjusting for baseline exposure to transgender health education, the interaction odds ratio rose to 2.11 (SE = 1.22; *p* = 0.07), suggesting that, when accounting for those two covariates, residents who received the lecture were marginally more likely than controls to shift into a higher confidence category.

Lastly, in the unadjusted Difference-in-Differences OLS model, the difference-in-differences estimate for Knowledge Score was 1.34 (SE = 0.70; *p* < 0.10), indicating that lecture attendees increased their total knowledge score by 1.34 points more than controls—a marginally significant effect. After adjusting for baseline exposure covariates, this estimate attenuated to 0.72 (SE = 0.75; *p* < 0.10), suggesting a smaller, still marginally positive shift among treated residents.

## Discussion

The study sought to determine whether an educational curriculum dedicated to identifying and addressing the healthcare needs of TGD individuals would positively influence residents’ knowledge and attitudes regarding transgender health. Similar studies addressing internal medicine resident education in LGBT health showed a positive correlation between curriculum participation and residents’ knowledge and self-confidence to address the health needs of TGD individuals [16, 17]. Our study attempted not only to address the need for more training on transgender health and health disparities but also to study the effect of such training on residents’ general knowledge and awareness of existing disparities. The controlled before-and-after design of the study allowed for a comparison with the results in a group of residents from one of the source programs who had not participated in the curriculum. In addition to the lack of significant change in the control group scores, the observed effect sizes were smaller within the categories of Knowledge and Self-Confidence. Furthermore, the regression analysis performed demonstrated improvements in knowledge and confidence in the study group that were specifically attributable to the lectures. This lends more confidence to the association between curriculum exposure in the study group and improvements in these domains.

A higher percentage of residents in the study group had received transgender health education before residency when compared with the control group, and we considered the possibility of prior education acting as a confounder. In that case, we would have expected higher pre-intervention Knowledge scores in the study group, but the average scores pre-intervention were similar between the two groups. Another possibility is that the findings were driven by individuals in the study group who were already interested in this topic and were thus more inclined to participate in the surveys, apart from the financial incentive. In that case, we would have expected self-selection bias to have a more pronounced effect than the one observed.

We developed an assessment tool to measure residents’ knowledge, confidence, and awareness of barriers to care faced by TGD individuals. As part of our assessment, we adapted items from the SOCCS, a validated tool designed to probe mental health providers’ understanding of challenges facing the TGD population and improve general preparation to work with TGD patients. To our knowledge, this is the first use of the SOCCS to evaluate the effects of an intervention at the graduate medical education level. We found positive correlations between curriculum exposure and improvements in self-confidence measures, but not in residents’ knowledge of barriers encountered by the TGD population, despite one objective of the study being to increase residents’ awareness of health inequities facing TGD individuals. No change was seen in either the study or control group in this domain. The fact that both groups began with a similar moderately high score at baseline may reflect a prevailing awareness of health disparities faced by TGD individuals. Detecting a change in this domain may require a different survey strategy.

A longitudinal curriculum was designed for the study to fill a gap in the literature on TGD health-specific education at the graduate level. Previous work done at the graduate level featuring one-time interventions addressing the TGD population led to short-term increases in knowledge and comfort that did not endure after several months [19, 21]. Other programs aimed at longitudinal education, such as the Transgender SCAN-ECHO program developed within the Veterans Affairs medical care system, have demonstrated significant improvements in providers' knowledge and comfort in TGD care [31]. The extended duration of our curriculum can be viewed as a strength in that exposure correlated with sustained effects in the knowledge and self-confidence domains, suggesting that repeated exposure to lecture topics on TGD health can produce more durable effects on resident knowledge and attitudes.

### Limitations and future research

The curriculum was directed toward improving residents’ knowledge of fundamental aspects of gender affirming care and their ability to approach TGD individuals in a culturally sensitive manner. As such, the lectures were not able to address all aspects of TGD health, including the higher prevalence of cardiovascular and other chronic conditions recognized in the TGD population [32, 33]. We also implemented the lecture series prior to the publication of updates in key guidelines including the WPATH Standards of Care Version 8 [34]. Further work will be needed both to provide training in the growing field of TGD health and research its impact.

The Transgender Assessment that we developed for the study comprised newly created multiple-choice knowledge questions and portions adapted from the SOCCS that were meant to query residents’ self-confidence and knowledge of barriers to care faced by the TGD population. We established face validity by having the survey reviewed by subject matter experts and a liaison from the TGD community, but we did not perform confirmatory factor analysis or collect representative data from our focus group samples. Comprehensive validation would thus be necessary before applying the survey in a broader study setting. Nonetheless, the survey detected significant differences between pre- and post-intervention responses and in the study and control groups, particularly in the Knowledge and Self-confidence domains.

Despite using financial incentives to encourage survey participation, a significant drop in post-intervention responses occurred in both groups over the 4-month period during which the curriculum took place. The smaller post-intervention sample size was particularly pronounced in the control group and likely limited the power to detect an effect in the Knowledge of Barriers domain in both groups. Other limitations of our study included the inability to infer causality due to the lack of paired pre- and post-intervention responses and the relatively short duration of the follow-up period.

Further educational development focused on the health of TGD individuals is needed at the graduate level [22]. The lack of a significant change in the Knowledge of Barriers domain pre- and post-intervention in either group suggests a need for further investigation into the appropriate educational strategies and methods for evaluating learners’ understanding of the health inequities facing the TGD population. Since one aim of health disparities education is greater cultural sensitivity, practice-based methods such as clinical rotations or simulations involving standardized patients may be necessary to discern the impact of training. Prior qualitative work involving TGD educators and activists has demonstrated the effectiveness of first-person accounts to convey the power of providers to act as barriers or facilitators to care for TGD patients [35]. While our didactics included content from TGD representatives, such content may need greater emphasis in a curriculum to result in a measurable effect on learners’ awareness of their own role in mediating health disparities.

## Conclusions

A curriculum focused on the health of TGD individuals can succeed in improving the knowledge and self-confidence of internal medicine residents to provide care to a population with major health disparities and who are often marginalized within society. Further study is needed into the durability of the effects seen and the ability of such curricula to improve awareness of the barriers to care faced by TGD individuals. Ultimately, we will need to know if educating our residents using both didactic and practice-based methods will translate into improved physical and mental health outcomes in this population.

## Supplementary Information

Supplementary Material 1.

Supplementary Material 2.

## Data Availability

Data is provided within the manuscript. The datasets and materials used are available from the corresponding author upon reasonable request.

## Abbreviations

FQHC: Federally qualified health center
LGBT: Lesbian, gay, bisexual, and transgender
OLS: Ordinary Least Squares
PGY: Post-graduate year
RUHS: Riverside University Health System
SD: Standard deviation
SE: Standard Error
SOCCS: Sexual Orientation Counselor Competency
TGD: Transgender and gender-diverse
USD: United States dollar
WPATH: World Professional Association for Transgender Health
