# Correction: Developing health care provider knowledge, confidence, and cultural sensitivity through resident transgender training: a controlled educational study

**DOI:** 10.1186/s12939-025-02602-3

**Published:** 2025-08-11

**Authors:** Kathie Huang, Almira J. Yang, Lynnetta Skoretz, Anthony Firek, Dhruv Khurana

**Affiliations:** 1 Department of Medicine, Riverside University Health System, 26520 Cactus Avenue, Moreno Valley, CA 92555 USA; 2 Department of Medicine, University of California Riverside School of Medicine, Riverside, CA USA; 3 Department of Medicine, Loma Linda University School of Medicine, Loma Linda, CA USA; 4 Department of Psychiatry and Biobehavioral Sciences, University of California Los Angeles David Geffen School of Medicine, Los Angeles, CA USA; 5 Department of Health Policy and Management, University of California Los Angeles Fielding School of Public Health, Los Angeles, CA USA; 6 Comparative Effectiveness and Clinical Outcomes Research Center (CECORC), Riverside University Health System, Moreno Valley, CA USA

**Correction: Int J Equity Health 24,** **202 (2025)**

**https://doi.org/10.1186/s12939-025-02555-7**

Following publication of the original article [1], an inadvertent omission was identified in the caption for Table 5. In the caption for Table 5, the bolded text has replaced “insert the two statements”.

Table 5: Represents the beta coefficients for the interaction term obtained from unadjusted and adjusted regression models based on the outcome variable as mentioned in the second column. The adjusted coefficients account for the two binary variables (**"I received some transgender health education through exposure outside of the classroom (e.g., news and social media outlets)” and “I received some transgender health education in the course of my education leading up to residency”**) that were statistically significantly different between the study and control groups at baseline

The original article [1] has been corrected.
