# Supplementary material for: Developing health care provider knowledge, confidence, and cultural sensitivity through resident transgender training: a controlled educational study
Source: Int J Equity Health. 2025 Jul 10;24:202. doi: 10.1186/s12939-025-02555-7 (PMC12247190; doi:10.1186/s12939-025-02555-7)
Supplement: Supplementary file 1 — Supplementary Material 1. [file 12939_2025_2555_MOESM1_ESM.docx]

Appendix 1.1: Inter-group Differences between Study and Control Group Demographics and Background

Appendix 1.2: Inter-group Differences between Study and Control Group Knowledge Responses

Appendix 1.3: Inter-group Differences between Study and Control Group Knowledge of Barriers Responses

Appendix 1.4: Inter-group Differences between Study and Control Group Self-Confidence Responses
