# Supplementary material for: Developing health care provider knowledge, confidence, and cultural sensitivity through resident transgender training: a controlled educational study
Source: Int J Equity Health. 2025 Jul 10;24:202. doi: 10.1186/s12939-025-02555-7 (PMC12247190; doi:10.1186/s12939-025-02555-7)
Supplement: Supplementary file 2 — Supplementary Material 2. [file 12939_2025_2555_MOESM2_ESM.docx]

**RUHS Transgender Assessment**

*In this survey, the following terms apply:*

*The term “transgender man” or “transgender male” refers to men whose sex was assigned as female at the time of their birth.*

*The term “transgender woman” or “transgender female” refers to women whose sex was assigned as male at the time of their birth.*

*The term “cisgender man” or “cisgender male” refers to men whose sex was assigned as male at the time of their birth.*

*The term “cisgender woman” or “cisgender female” refers to women whose sex was assigned as male at the time of their birth.*

*The term “transgender health education” refers to training on aspects of prevention and treatment for transgender individuals, including gender affirming medical and surgical therapies.*

Baseline Questions

Age __

PGY training program level

Gender that I identify as:

Male / Female / Non-Binary / Genderqueer / Other ___ / Decline to answer

Sex assigned at birth:

Male / Female / Decline to answer

Sexual orientation:

Straight / Lesbian / Gay / Bisexual / Other _____ / Decline to answer

Yes/No

I identify as transgender.

I personally know someone who identifies as transgender.

I have recently (in the past 1 year) interacted with a transgender person in the community (outside of the medical setting).

I received some transgender health education in the course of my education leading up to residency.

I received some transgender health education through exposure outside of the classroom (e.g. news and social media outlets).

I have previously received adequate training about transgender health care.

Yes/No

I have experience providing care to transgender female patients.

Yes/No

I have experience providing care to transgender male patients.

Yes/No

My experiences with transgender individuals have positively affected my thoughts about what it means to be transgender (if you have not met a transgender individual, select “not applicable”)

1 strongly disagree, 2 Moderately disagree, 3 Neither agree nor disagree 4 Moderately agree, 5 strongly agree OR Not applicable

Knowledge Section

1 – Gender affirming medical and surgical therapies

2 – Screening and preventive care

3 – Sexual health

**Correct answers are bolded**

Fill in the blank with the best choice below (1)

______________ is/are indicated treatment for most transgender patients and should be provided to patients who request them if there is no contraindication.

**A) Hormone and surgical therapies**

B) Only hormone therapy

C) Only surgical therapy

D) Neither hormone nor surgical therapy

Choose the best answer. Gender affirming hormone treatment: (1)

A) Should be prescribed to transgender patients once they report gender dysphoria defined as distress over incongruence with their gender identity and the sex they were assigned at birth

**B) Should be offered to transgender patients who request it after carefully evaluating the risks and benefits**

C) May result in unacceptable side effects to the degree that it should not be offered

D) Is not appropriate treatment for gender dysphoria as the person should primarily be offered mental health treatment

During male-to-female sex reassignment surgery, the ____________ is NOT surgically manipulated or removed. (1)

**A) Prostate gland**

B) Glans penis

C) Scrotum

D) Vas deferens

A 55-year-old transgender man who has been treated with gender affirming hormones for 5 years but has not undergone sex reassignment surgery should receive all of the following EXCEPT: (2)

A) Breast cancer screening

**B) Prostate cancer screening**

C) Cervical cancer screening

D) Colon cancer screening

A 44 year old transgender woman has been treated with gender affirming hormones since the age of 42. Breast cancer screening for the patient should begin: (2)

A) At the age of 50 because the incidence of breast cancer in transgender females is the same as in cisgender females

**B) At the age of 50 with the absolute risk of breast cancer in transgender females being below that of the cisgender female population**

C) Now because of the observed association between hormone replacement therapy and the increased risk of breast cancer in cisgender females

D) At the age of 45 after a minimum exposure time of 3 years since screening is recommended according to the length of time exposed to estrogens

A 26 year-old transgender man presents for follow-up. He has been on stable testosterone therapy for 12 months and not yet undergone gender affirming surgery. He reports to you that he is currently sexually active with his cisgender male partner and engages in penetrative vaginal sex. What is the best way to counsel him on contraception? (3)

A) Testosterone therapy serves as an adequate method of contraception

B) Condom use is the only safe contraceptive method for him

C) The patient should not use a progesterone subdermal implant

**D) Either condoms or a progesterone subdermal implant can be effective contraceptive options for the patient**

A 34 year old transgender man reports to his primary care physician that he has been taking testosterone that he obtained independently from a non-medical source. After counseling the patient about the importance of appropriate dosing and monitoring of testosterone therapy, the physician agrees to prescribe the patient testosterone. The physician’s decision is best described as being based on the principle of (1):

A) Cultural humility

**B) Harm reduction**

C) Respect for autonomy

D) Trauma informed care

A 23 year old patient who was assigned male at birth reports coming out as a trans woman. She is interested in exploring gender affirming hormone treatment to achieve greater physical congruence with her gender identity. Which of the following statements is most appropriate in counseling the patient? (1)

A) Gender affirming hormone treatment is reserved for transgender patients who are experiencing gender dysphoria

B) Transgender patients should undergo consultation with a psychologist or psychiatrist prior to starting hormone treatment

C) It is necessary to evaluate her for a disorder of sexual development by performing a karyotype prior to proceeding with hormone treatment

**D) An informed consent process that takes into account the patient’s full medical and mental health history should occur before proceeding with hormone treatment**

A 25 year old transgender woman who is sexually active with cisgender men presents for testing and treatment. Her last sexual encounter was 5 weeks ago with a new partner who informed her last week that he had been diagnosed and treated for syphilis. Her only medication currently is estrogen. The patient receives a rapid HIV test in the clinic that is negative. In addition to screening her for sexually transmitted infections and hepatitis, which of the following is the most appropriate next step? (3)

A) Inform the patient that she is eligible for HIV pre-exposure prophylaxis (PrEP) but that her dosage of estrogen must be reduced to minimize drug-drug interactions

**B) Inform the patient that she is at a high risk for HIV acquisition and her risk can be most effectively reduced by taking PrEP**

C) Inform the patient that the most effective way to reduce her risk of acquiring HIV is to achieve a mutual agreement with her partners to use condoms with every sexual encounter

D) Inform the patient that she does not need to take PrEP if she can ascertain the HIV negative status of her future partners

Knowledge of Barriers Section

Response scale:

1 Strongly disagree, 2 Moderately disagree, 3 Neither agree nor disagree 4 Moderately agree, 5 Strongly agree

Transgender patients receive clinical treatments inferior to treatments offered to non-transgender patients when their providers lack medical knowledge about transgender health care.

Transgender people often face discrimination and stigmatization due to biases held by healthcare professionals.

Institutional barriers exist that may inhibit transgender people from using healthcare services.

Healthcare providers often impose their values concerning gender on transgender patients which can lead to suboptimal care.

Self-Confidence Section

Response scale:

1 Strongly disagree, 2 Moderately disagree, 3 Neither agree nor disagree 4 Moderately agree, 5 Strongly agree

At this point in my training, I feel confident to assess the medical needs of transgender patients.

At this point in my training, I feel confident to provide gender health-specific care to transgender patients.

I feel comfortable communicating with transgender patients.

When a patient who was assigned female at birth reports identifying as a male since adolescence and requests gender affirming hormonal therapy, I feel confident assisting with this.

When a patient who was assigned male at birth reports identifying as a female since adolescence and requests gender affirming hormonal therapy, I feel confident assisting with this.
